# Higher plasma betatrophin/ANGPTL8 level in Type 2 Diabetes subjects does not correlate with blood glucose or insulin resistance

**DOI:** 10.1038/srep10949

**Published:** 2015-06-16

**Authors:** Mohamed Abu-Farha, Jehad Abubaker, Irina Al-Khairi, Preethi Cherian, Fiona Noronha, Frank B. Hu, Kazem Behbehani, Naser Elkum

**Affiliations:** 1 Biochemistry and Molecular Biology Unit; 2 Biostatistics and Epidemiology Department Dasman Diabetes Institute, Kuwait City, Kuwait; 3 Departments of Nutrition and Epidemiology, Harvard School of Public Health, Boston, Massachusetts, United States of America; 4 Sidra Medical and Research Center, Qatar

## Abstract

Betatrophin/ANGPTL8 is a newly identified hormone produced in liver and adipose tissue that has been shown to be induced as a result of insulin resistance and regulates lipid metabolism. Little is known about betatrophin level in humans and its association with T2D and metabolic risk factors. Plasma level of betatrophin was measured by ELISA in 1603 subjects: 1047 non-diabetic and 556 T2D subjects and its associations with metabolic risk factors in both non-diabetic and T2D were also studied. Our data show a significant difference in betatrophin levels between non-diabetic (731.3 (59.5–10625.0) pg/ml) and T2D (1710.5 (197.4–12361.1) p < 0.001. Betatrophin was positively correlated with age, BMI, waist/hip ratio, FBG, HbA1C, HOMA-IR and TG in the non-diabetic subjects. However, no association was observed with BMI, FBG, HbA1C or HOMA-IR in T2D subjects. TC and LDL showed negative association with betatrophin in T2D subjects. Multivariate analysis showed that subjects in the highest tertile of betatrophin had higher odds of having T2D (odd ratio [OR] = 6.15, 95% confidence interval [CI] = (3.15 – 12.01). Our data show strong positive associations between betatrophin and FBG and insulin resistance in non-diabetic subjects. However, correlations with FBG and insulin resistance were diminished in T2D subjects.

Type 2 Diabetes (T2D) is a major burden to human health, affecting over 336 million people worldwide^1^. While autoimmune system destruction of β-cells is the main cause of β-cell loss in type 1 diabetes (T1D)^2^,^3^,^4^, the failure of β-cells to compensate for ambient insulin resistance leads to uncontrolled hyperglycemia in T2D^1^,^5^,^6^,^7^,^8^. Even though glycemic control can be achieved through the use of various types of anti-diabetic drugs including insulin injection, full glycemic control similar to functional β-cells is hard to achieve^9^. Alternatively, enhancing the proliferation of β-cells in the pancreas can lead to better treatments for T2D^10^. Existing inducers of insulin secretion or β-cell replication such as glucagon-like peptide 1 (GLP-1) and gastric inhibitory polypeptide (GIP) are not specific to this type of cells and only cause a small increase in β-cell proliferation^7^. A novel protein called betatrophin has been recently found to increase the beta-cell mass in an insulin resistance mouse model^9^. To do so, Yi *et al.*, generated an insulin resistant mouse model using an insulin receptor antagonist and showed that betatrophin was up-regulated in the liver and fat tissues. It’s over expression was shown to cause a 17-fold increase in β-cell proliferation and to increase β-cell mass in mice^9^.

Betatrophin is yet another name given to C19orf80, which is also called Hepatocellular Carcinoma-Associated Gene TD26. Ren *et al.*, gave betatrophin the name RIFL (refeeding induced in fat and liver) as they demonstrated that betatrophin was induced in 3T3 L1 cells during adipogenesis and its knockdown led to a reduction in adipogenesis^11^. Betatrophin was also given the name angiopoietin-like 8 (ANGPTL8) as it shares significant sequence similarity with the ANGPTL protein family particularly ANGPTL3. Betatrophin and ANGPTL3 were shown to interact at the protein level affecting levels of triglycerides (TG), high density lipoprotein cholesterol (HDL-C) and low density lipoprotein cholesterol (LDL-C)^12^. In addition, Quagliarini *et al.*, showed that a non-synonymous variant in betatrophin that changes amino acid 59 from Arginine (R) to Tryptophan (W) (R59W) was associated with lower HDL-C and LDL-C levels in human plasma^12^. Due to its activity in lipoprotein lipase inhibition, betatrophin was also called Lipasin; its expression was induced by cold shock at 4 °C and a high fat diet, while fasting reduced its expression^13^,^14^.

Liver produced hormones have been suggested as potential mediators of the increased β-cell proliferation under an insulin resistant state^15^,^16^. Betatrophin has been suggested as another example of a liver derived hormone that is capable of inducing β-cell proliferation by Yi *et al*^9^. They have suggested that betatrophin levels are regulated by insulin resistance and not insulin deficiency in mice, suggesting a strong role for this protein in T2D^9^. However, this work has been disputed in a recent article by Gusarova *et al.* which showed that mice lacking betatrophin/ANGPTL8 had a normal β-cell expansion under states of insulin resistance induced by high-fat diet or treatment with insulin receptor antagonist S961^17^. In response, Yi *et al.* repeated their experiments and failed to replicate their earlier experiment concluding that betatrophin does not regulate β-cell expansion^18^. Recent reports have shown that betatrophin was increased in T2D^19^,^20^ while others suggested that betatrophin was reduced in subjects with T2D^21^. To address this question, this study evaluated the association of betatrophin with blood sugar and IR in subjects with-and-without T2D in a large sample set.

## Research Design and Methods

### Study Participants

In order to understand the role of betatrophin in T2D, plasma samples from 1603 adult (>18 years old) South Asians and Arabs living in Kuwait were used. This is part of a large cohort that has been randomly collected from multi-ethnic subjects living in Kuwait as described previously^22^,^23^,^24^. Briefly, a stratified random sampling technique was used for the selection of participants from the computerized register of the Public Authority of Civil Information. This survey was carried out between June 2011 and August 2012. A total of 3468 subjects randomly selected across the six governorates (strata) of Kuwait were collected. 722 subjects were diabetics. A total of 556 T2D subjects were used in this study after excluding subjects with T1D and cardiovascular diseases. Study participants suffering from any kind of infection as well as subjects younger than 18 and older than 65 were excluded. Controls were then selected as subjects without disease and not taking any medications, as a result, a total of 1047 non-diabetic subjects were selected. The selected non-diabetic subjects had similar population characteristics as the total study population in terms of age, gender, FBG, BMI, BP and lipid profile. The study conformed to the principles outlined in the Declaration of Helsinki and in accordance with the approved guidelines. The study was approved by the Scientific Advisory Board and Ethical Review Committee at Dasman Diabetes Institute (DDI). An informed written consent was obtained from all the participants before their enrolment in the study.

### Anthropometric and Physical Measurements

Physical and anthropometric measurements taken were: body weight, height, waist circumference (WC) and BP. BP was measured using an Omron HEM-907XL Digital sphygmomanometer. An average of 3 BP readings, with 5 to 10 minutes rest between each, was obtained. Height and weight were measured, with participants wearing light indoor clothing and barefooted, using calibrated portable electronic weighing scales and portable inflexible height measuring bars. WC was measured using constant tension tape at the end of a normal exhalation, with arms relaxed at the sides, at the highest point of the iliac crest and at the mid-axillary line. BMI was calculated using the standard BMI formula: body weight (in kilograms) divided by height (in meters squared).

### Laboratory Measurements

Blood samples were obtained after fasting overnight for at least 10 hours and analyzed for FBG, HbA1c, fasting insulin, and lipid profiles that included TG, TC, LDL and HDL. Glucose and lipid profiles were measured on the Siemens Dimension RXL chemistry analyzer (Diamond Diagnostics, Holliston, MA). HbA1c was determined using the VariantTM device (BioRad, Hercules, CA). All laboratory tests were performed by certified technicians at the clinical laboratories of DDI using the Ministry of Health approved methods and quality standards. Insulin was measured using the Access Ultrasensitive Insulin Assay (Beckman Coulter, Brea, CA). Intra- and intra- assay coefficients of variation were ≤6%. Insulin resistance was calculated using HOMA-IR formula: FBG (mmol/L) x fasting insulin (mU/L) / 22.5. HOMA-β was calculated using the following formula (20 x fasting insulin (mU/L) / FBG (mmol/L)-3.5)*100%.

### Diabetes diagnosis and guidelines

The current recommendations and updated guidelines for the definition, diagnosis and classification of T2D, published by the International Diabetes Federation (IDF), have been used 25. Diabetes was defined by fasting plasma glucose ≥7 mmol/l, under treatment, or self-reporting of previously diagnosed T2D^25^. Impaired fasting glucose (IFG) was defined by fasting blood glucose values ≥5.6 and <7 mmol/L.

### ELISA Betatrophin and Level

Blood samples were drawn into vacutainer EDTA aprotinin tubes. Plasma was obtained after centrifugation for 10 minutes at 2000 g at room temperature. Plasma was then aliquoted into cryogenic tubes and stored at −80 °C. Plasma samples stored at −80 °C freezers were thawed on ice and centrifuged at 10000 g for 5 minutes at °C 4 to remove any debris. Samples used to measure level of betatrophin were not exposed to freeze thaw cycles. Betatrophin concentration was determined using ELISA (Wuhan EIAAB Science co) as described previously^26^,^27^. Current ELISA kit was validated against other available kits showing correlation coefficient of 0.99^28^. Furthermore, we have validated the current kit using recombinant betatrophin. A range of betatrophin concentrations were used to spike in plasma at different dilution factors. The assay showed linearity at dilutions ranging from 1:10–1:40. Recovery of the known proteins ranged from 85–109%. No significant cross reactivity with other proteins was observed. Intra-assay coefficients of variation were 1.2% to 3.8%, while the inter-assay coefficients of variation were 6.8% to 10.2%.

### Statistical Analysis

Normality tests were used to assess data distribution. Comparisons between subjects with T2D and without T2D were made by Student’s t-test or Wilcoxon test for non-parametric analyses in variables with non-normal distribution. To assess the difference in categorical variables between subjects with and without T2D, a Chi-Squared test was used. Spearman’s correlation coefficients were estimated to determine associations between betatrophin and anthropometric measurements and biochemical variables. Subjects were classified into tertiles based on their circulating betatrophin levels in the overall population. Tertile values of betatrophin are expressed as T1 (<700 pg/mL), T2 (700 – 1287.5 pg/mL), and T3 (> 1287.5 pg/mL). A multivariable logistic regression analysis was performed to estimate odds ratios (ORs) adjusted for covariates and to assess the predictive effect of betatrophin on risk for T2D. All data are reported as Mean ± Standard Deviation (SD) and range, unless stated otherwise. Research Electronic Data Capture (REDCap) was used for data collections and data management. All statistical assessments were two-sided and considered to be significant when *P-value* <0.05. All analyses were performed using SAS (ver sion 9.2; SAS Institute, Cary, NC).

## Results

Characteristics of the study population are outlined in Table 1. Our population was made of 1603 subjects, 1047 of which were non-diabetic and 556 were T2D. The average age of participants was 42.5 ± 10.3 for non-diabetic subjects and 53.2 ± 9.7 for subjects with T2D. Subjects with T2D had a significantly higher BMI, waist/hip ratio, systolic BP, diastolic BP, FBG, HBA1C, insulin, HOMA-IR, TG, TC, HDL, and LDL (p < .05). Betatrophin showed more than a two fold increase in subjects with T2D (1710.5 (197.4 – 12361.1) pg/mL) relative to non-diabetic subjects (731.3 (59.5 – 10625.0) pg/mL).

Using Spearman’s correlation adjusted for gender and ethnicity betatrophin was positively associated with age (*r* = 0.49, *p* = <0.0001), BMI (*r* = 0.14, *p* = <0.0001), waist/hip ratio (*r* = 0.16, *p* = <0.0001), FBG (*r* = 0.19, *p* = <0.0001), HbA1c (*r* = 0.14, *p* = <0.0001) insulin (*r* = 0.14, *p* = <0.0001), HOMA-IR (*r* = 0.17, *p* = <0.0001) and TG (*r* = 0.15, *p* = <0.0001) in the non-diabetic subjects (Table 2). In the T2D subjects betatrophin only showed significant correlation with age (*r* = 0.46, *p* = <0.0001), waist/hip ratio (*r* = 0.18, *p* = <0.0001) and duration of T2D (*r* = 0.29, *p* = <0.0001). It also showed negative correlation with TC (*r* = −0.25, *p* = <0.0001) and LDL(*r* = −0.27, *p* = <0.0001). No correlation was observed with BMI, FBG, HbA1C and HOMA-IR or HOMA-β (Table 2).

**Figure 1:**
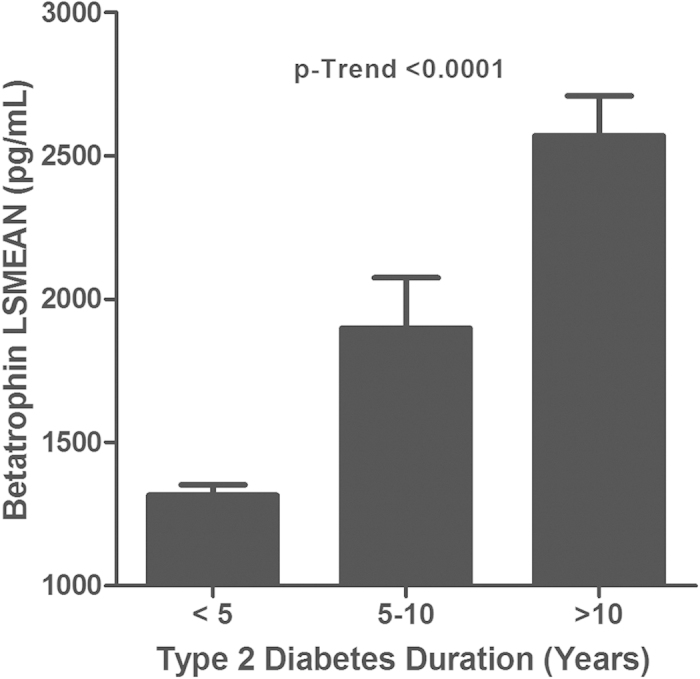
Age-, gender- and ethnicity-adjusted least square mean estimate of betatrophin in T2D patients plotted against duration of T2D.

As shown by Spearman’s correlation (Table 2), betatrophin levels were associated with increased duration of T2D. Stratifying T2D patients according to duration of the disease showed that people with a longer duration of diabetes had higher betatrophin levels. Subjects with T2D for less than 5 years had the least square means of betatrophin, 1315.5 (38.1–74.5) pg/mL, compared to 1898.3 (176.7–346.3) pg/mL for subjects that had T2D for 5–10 years and 2569.6 (139.0–272.3) pg/mL for subjects who had T2D for longer than 10 years (Fig. 1).

After adjusting for age, gender and ethnicity (model 1), subjects in the highest tertile of betatrophin were more likely to have T2D (OR = 10.94, 95% CI = 7.29 – 16.42) (Table 3). After additional adjustment for BMI and waist/hip ratio, the results did not materially change. Compared to subjects in the lowest tertile of betatrophin, those in the highest tertile had higher odds of having T2D (OR = 10.09, 95% CI = 6.68 – 15.24). Further adjustment for HbA1c, HOMA-IR and HOMA-β moderately attenuated the association, but subjects in the highest tertile still had higher odds of having T2D (OR = 7.37, 95% CI = 3.87 – 14.04) (*p*-trend <0.0001) (Table 3). Adjusting for total cholesterol, triglyceride, LDL, HDL, systolic, diastolic blood pressure + Model 3 further attenuated the association showing that subjects in the highest tertiles of betatrophin had higher odds of having T2D (OR = 6.15, 95% CI = 3.15 – 12.01) (*p*-trend <0.0001).

**Figure 2:**
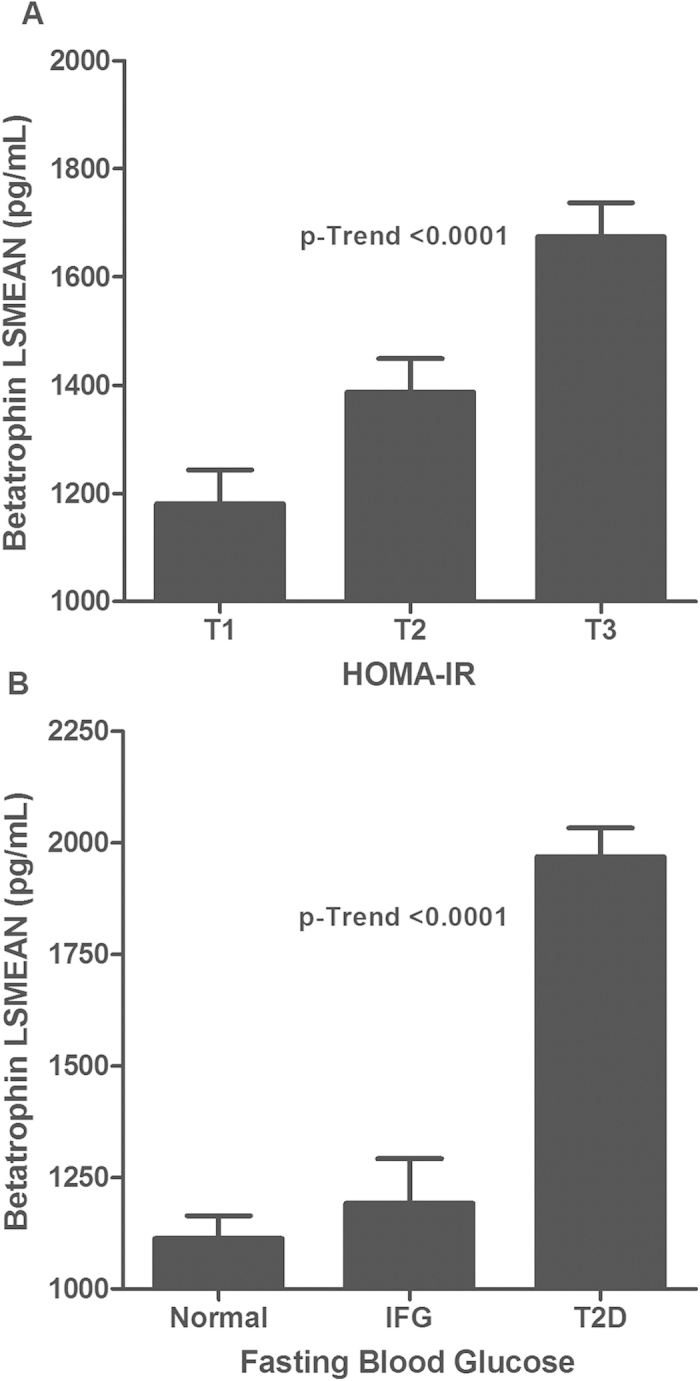
Betatrophin level distribution according to insulin resistance and FBG. **A**: Age-, gender-, and ethnicity-adjusted least square means of concentrations of betatrophin according to HOMA-IR. Tertile values of HOMA-IR are expressed as T1 (≤1.6), T2 (>1.6 & ≤3.1), and T3 (>3.1). **B**: Age-, gender-, and ethnicity-adjusted least square means of concentrations of betatrophin according to FBG and HOMA-IR. T2D: FBG ≥7 mmol/l, under treatment, or self-reported of previously diagnosed T2D; IFG: fasting blood glucose values ≥5.6 and <7 mmol/L. LSMean: Least Square Mean.

Multivariate regression analysis showed that FBG and HOMA-IR were significantly associated with higher betatrophin levels (Fig. 2). Age-, gender- and ethnicity-adjusted least square means of concentrations of betatrophin, according to FBG and HOMA-IR are shown in Fig. 2. Data presented in Fig. 2A shows the association between increased insulin resistance and increasing levels of betatrophin (*p*-trend <0.0001). Figure 2B shows that increased betatrophin levels were significantly associated with increased FBG levels (*p*-trend <0.0001).

## Discussion

In this study, we have used a large sample size to look at the level of betatrophin in 1047 non-diabetic and 556 T2D subjects. Our data clearly demonstrated an increase in betatrophin level in T2D compared to non-diabetic. Betatrophin level showed strong correlation with age in older subjects in both T2D and non-diabetic subjects. However, correlations with BMI, FBG, TG and HOMA-IR were only observed in non-diabetic subjects. In T2D subjects, betatrophin showed significant positive correlation with waist/hip ratio and negative correlation with LDL and TC. Betatrophin levels showed significant increase in subjects with longer duration of T2D. Finally, higher betatrophin levels were associated with more than six-fold increase in the odds of having T2D after adjustment for a wide range of risk factors.

Recent reports investigating betatrophin link to T2D circulation showed contradictory data. A number of studies showed that betatrophin level was increased in T2D subjects^19^,^20^,^21^ while, Gomez-Ambrosi *et al.* showed that betatrophin was reduced in T2D subjects^29^. In the current study, we showed that circulating levels of betatrophin were increased in subjects with T2D compared to subjects without T2D. This increase may be related to increased insulin resistance and higher demand for insulin in T2D subjects. Our data agrees with data reported in mice showing that betatrophin levels were up-regulated by 3–4 folds at the transcription level in the liver of the db/db and ob/ob mice models compared to wild-type^9^. This trend in increased betatrophin levels were also observed in patients with longer duration of T2D in our population as shown in Fig. 1. More recent reports have also showed that betatrophin was increased in T2D and T1D^19^,^20^,^21^,^26^. However, a recent report found that betatrophin level was reduced in T2D^29^. Fu *et al.*, suggested that the discrepancies were caused by different betatrophin species that were measured using different ELISA kits^28^. Even though this represents a good possibility, differences in sample size and ethnic groups across studies may also contribute to different findings^19^,^20^,^21^,^29^.

Age was another factor that positively correlated with betatrophin levels in both T2D and non-diabetic subjects. A similar trend was observed in other studies^21^,^26^,^27^. Espes *et al.* showed that betatrophin levels were higher in older non-T1D control individuals, while T1D patients did not show a similar trend^26^. In contrast, T2D patients in our population showed an age dependent increase in their betatrophin levels. The difference between the two types of diabetes is interesting and supports a possible functional importance of betatrophin in T2D, where it could be playing a role in compensating for the increased insulin demand. This is in agreement with previous reports which highlighted the attenuated induction of β-cell proliferation in older mice when compared to young mice^30^ and in adult human β-cells compared to embryonic and neonatal β-cells^31^,^32^. However, the lack of association between betatrophin level and FBG and HOMA-IR in diabetic subjects raises serious concern about the functional use of betatrophin especially in light of recent reports showing that human β-cells were not responsive to the increase in betatrophin level^33^. In addition, the recent critical paper by Gusarova *et al.* showed that β-cell expansion under insulin resistance conditions was not affected by the deletion of both copies of betatrophin^17^. Nonetheless, the increased level of betatrophin in T2D subjects is interesting and further raises the question about the actual function of betatrophin especially after the recent reports confirming that betatrophin does not affect the beta cell expansion in mice^17^,^18^ and humans^33^. More studies will be required to answer these questions and better establish the cellular role of betatrophin in T2D.

In addition to using a large sample set, our study focused on a high risk group where recent epidemiological data from Arabian Gulf countries warned of the high prevalence of obesity and T2D^34^,^35^,^36^,^37^. According to International Diabetes Federation (IDF) Bahrain, Kuwait, Oman, Saudi Arabia, and United Arab Emirates ranked amongst the highest countries in the world with T2D^35^,^37^. Asians on the other hand have a higher risk for hypertension, T2D, cholesterol profiles and cardiovascular disease^38^. One of the main limitations of the current study is the cross sectional nature that cannot establish causality. In conclusion, we showed that betatrophin level was increased in T2D subjects albeit it did not correlate with FBG or insulin resistance in these patients. On the other hand, betatrophin levels were significantly correlated with FBG and insulin resistance in non-diabetic subjects. The increased betatrophin levels with age in both T2D and non-diabetic subjects can potentially represent a possible compensatory cellular mechanism in older subjects. More studies will be required to determine any causal relationship between betatrophin and T2D.

## Additional Information

**How to cite this article**: Abu-Farha, M. *et al.* Higher plasma betatrophin/ANGPTL8 level in Type 2 Diabetes subjects does not correlate with blood glucose or insulin resistance. *Sci. Rep.* **5**, 10949; doi: 10.1038/srep10949 (2015).

## Figures and Tables

**Table 1:** Clinical and biochemical profiles for non-diabetic and T2D subjects.

**Variables**	**Non-diabetic (n = 1047)**	**T2D (n = 556)**	***P*-value**
Age (years)	42.5 ± 10.3	53.2 ± 9.7	<0.0001
**Gender**			
Male	597(57.02%)	348(62.59%)	0.0310
Female	450(42.98%)	208(37.41%)	
**Ethnicity**			
Arab	596(56.92%)	371(66.73%)	0.0001
South Asian	451(43.08%)	185(33.27%)	
BMI (kg/m^2^)	29.31 ± 6.02	31.35 ± 5.84	<0.0001
Waist/hip ratio	0.91 ± 0.09	0.95 ± 0.06	<0.0001
Systolic (mmHg)	128.59 ± 18.42	138.43 ± 19.71	<0.0001
Diastolic (mmHg)	78.96 ± 12.03	80.98 ± 11.34	0.0008
FBG (mmol/L)	5.09 ± 0.57	8.97 ± 3.32	<0.0001
HBA1C (DCCT%)	5.51 ± 0.61	8.09 ± 1.97	<0.0001
Insulin (mU/L)	9.00 ± 5.55	14.35 ± 12.03	<0.0001
HOMAIR	2.08 ± 1.41	5.75 ± 5.34	<0.0001
Total cholesterol (mmol/L)	5.18 ± 1.06	4.95 ± 1.13	<0.0001
Triglycerides (mmol/L)	1.42 ± 0.81	1.88 ± 1.37	<0.0001
HDL cholesterol (mmol/L)	1.16 ± 0.33	1.08 ± 0.28	<0.0001
LDL cholesterol (mmol/L)	3.04 ± 0.95	3.40 ± 0.95	<0.0001
Subjects taking insulin injection		174 (31.29%	
Creatinine μmol/L	77.50 ± 18.18	81.92 ± 29.68	0.0014
Betatrophin (pg/mL)	731.3 (59.5 – 10625.0)	1710.5 (197.4 – 12361.1)	<0.0001

Results are reported as Mean ±SD except for non-normally distributed betatrophin that are presented as Median (interquartile range). Diabetes: fasting blood glucose ≥7 mmol/l, under treatment, or self-reported of previously diagnosed T2D; WC, waist circumference.

**Table 2:** Spearman correlations between betatrophin levels and T2D risk factors.

**Variables**	**Non-diabetic**		**T2D**	
**ρ**	***P*****-value**	**ρ**	***P*-value**	
Age (years)	0.49	<0.0001	0.46	<0.0001
BMI (kg/m^2^)	0.14	<0.0001	−0.04	0.3931
Waist/hip ratio	0.16	<0.0001	0.18	<0.0001
FBG (mmol/L)	0.19	<0.0001	−0.04	0.3162
HbA1C, %	0.14	<0.0001	0.02	0.6140
Insulin (mU/L)	0.14	<0.0001	−0.02	0.6680
HOMA-IR	0.17	<0.0001	−0.03	0.4776
HOMA-β	0.05	0.1526	0.01	0.7070
Total cholesterol (mmol/L)	0.05	0.0845	−0.25	<0.0001
Triglycerides (mmol/L)	0.15	<0.0001	−0.02	0.5714
HDL cholesterol (mmol/L)	−0.03	0.3995	0.001	0.9819
LDL cholesterol (mmol/L)	0.02	0.5426	−0.27	<0.0001
Diabetes duration (years)	-	-	0.29	<0.0001

BMI, FBG, T2D: fasting blood glucose ≥7 mmol/l, under treatment, or self-reported of previously diagnosed T2D. Duration of T2D was calculated for subjects with diabetes. Partial Spearman correlation coefficients were adjusted for age, gender, and ethnicity.

**Table 3:** Multiple logistic regression models for diabetes in relation to betatrophin.

	**T1, n = 537 T2D n = 45 Non-diabetic n = 492**	**T2, n = 532 T2D n = 149 Non-diabetic n = 383**	**T3, n = 534 T2D n = 362 Non-diabetic n = 140**	***P*-trend**
Betatrophin§ (pg/mL)	500.0 (59.5 – 700.0)	932.4 (701.4 – 1287.5)	2078.9 (1289.5 – 12361.1)	<0.0001
**Models**				
**T2D**	Reference	OR (95% CI)	OR (95% CI)	
Model 1	1	2.82 (1.92 – 4.14)	10.94 (7.29 – 16.42)	<0.0001
Model 2	1	2.66 (1.80 – 3.92)	10.09 (6.68 – 15.24)	<0.0001
Model 3	1	1.52 (0.82 – 2.79)	7.37 (3.87 – 14.04)	<0.0001
Model 4	1	1.46 (0.78 – 2.73)	6.15 (3.15 – 12.01)	<0.0001

Model 1 adjusted for age, gender, and ethnicity; Model 2 adjusted for BMI, waist/hip ratio + Model 1; Model 3 adjusted for HbA1c, HOMA-IR, and HOMA-β + Model 2. Model 4 adjusted for total cholesterol, triglyceride, LDL, HDL, systolic, diastolic blood pressure + Model 3. Tertile values of betatrophin are expressed as T1 (<700.0), T2 (700.0 – 1287.5), and T3 (>1287.5).

^§^Betatrophin presented as Median (range).
